# Modulation of the autophagic pathway inhibits HIV-1 infection in human lymphoid tissue cultured ex vivo

**DOI:** 10.1038/s41598-022-11181-0

**Published:** 2022-05-06

**Authors:** Sònia Pedreño-López, Elisabet García, Dolores Guerrero, Elisabet Gómez-Mora, Laura Molina Mateu, Fernando Orera Pérez, Jordi Senserrich, Bonaventura Clotet, Cecilia Cabrera

**Affiliations:** 1 AIDS Research Institute-IrsiCaixa and Health Research Institute Germans Trias i Pujol (IGTP), Hospital Germans Trias i Pujol, Universitat Autònoma de Barcelona, Carretera del Canyet S/N, 08916 Badalona, Barcelona Spain; 2 Otorhinolaryngology Department, Hospital Germans Trias i Pujol, Universitat Autònoma de Barcelona, 08916 Badalona, Spain; 3 Infectious Diseases Department, Hospital Germans Trias i Pujol, Badalona, Catalonia Spain; 4 Universitat de Vic Central de Catalunya, Vic, Catalonia Spain

**Keywords:** HIV infections, Macroautophagy

## Abstract

A complex link exists between HIV-1 and autophagy, and discordant results have been reported in different in vitro models regarding the way HIV and autophagy modulate each other. Despite this, there is very limited knowledge about the interplay between HIV and autophagy in vivo in lymphoid tissue, due in part by the lack of cell models that recapitulate the in vivo setting. Here, we evaluate the interrelationship between HIV and autophagy using human ex vivo lymphoid tissue cultures as an HIV infection model. Our results showed that human lymphoid aggregated cultures (HLACs) from tonsillar tissue displayed fully functional autophagic activity. In this system, HIV infection resulted in an increase in autophagy. Notably, we observed that both, autophagy-enhancing (rapamycin) or blocking drugs (3-methyladenine, chloroquine and bafilomycin), were able to decrease HIV-DNA levels and HIV replication. Therefore, efficient HIV-1 replication requires a fine-tuned level of autophagy, so modifications of this balance will have a negative impact on its replication. Therefore, targeting the autophagic pathway could be a new therapeutic approach to be explored to treat HIV-1 infection. Ex vivo cultures of human lymphoid tissue are a suitable model to obtain further insights into HIV and its intricate relationship with autophagy.

## Introduction

Autophagy has been shown to participate in a number of important cellular functions ranging from adaptation to starvation, cell differentiation and development, aging, turnover of superfluous or damaged organelles (Review in^1^) and innate and adaptive immunity against pathogens^2^. The most common approach to measure autophagy and the autophagic flux is by western blot, through microtubule-associated protein light chain 3 (LC3) protein detection and their conversion from LC3-I to LC3-II, as the amount of LC3-II is clearly correlated with the number of autophagosomes, the hallmark of autophagy.

Different effects of HIV-1 on autophagy have been described depending on the cell type and on the infectious status of the target cell type; i.e., if the target cell is productively infected or not^3,4^. In CD4^+^ T lymphocytes, it has been shown that autophagy is induced in uninfected bystander CD4^+^ T cells, using both primary and cell lines^5,6^, and blocked in productively infected MOLT-4 CD4^+^ T cells^7^. On the contrary, in other reports CD4^+^ T cell infection is associated with autophagic induction in both Jurkat and primay CD4^+^ T cells^8^. In productively infected macrophages or monocytic cells, an upregulation of autophagy is observed, while autophagy is blocked in uninfected bystander macrophages, although this effect is not confirmed by other authors^7,9–11^. Similar discordance has been reported in the effect of autophagy modulation in HIV-1 replication. Autophagy induction and inhibition has been associated with HIV-restriction, although an increase in viral replication has also been described^8,11–13^. Overall, the discrepancy between all these studies could stem from the different cellular models used (primary cells and cell lines) and the cellular state highlighting that autophagy is a cell type dependent process.

To date, relatively few studies have investigated the interplay of HIV and autophagy in vivo during HIV infection. In long-term non-progressors (LTNP), infected individuals that naturally control the viral replication in the absence of antiretroviral treatment, the resistance to HIV-1-induced pathogenesis is accompanied by a significant increase in the autophagic activity in peripheral blood mononuclear cells (PBMCs)^14^. Furthermore, an impairment in the autophagy induced response has been observed in HIV-1 individuals who fail to recover their CD4^+^ T cells after ART treatment^15^. In this study, autophagy was determined in CD4^+^ T cells by immunofluorescence in a group of aviremic-treated HIV^+^ individuals with either optimal of poor CD4 T-cell recovery and in healthy controls. The results showed that autophagy was significantly decreased in CD4 T cells from HIV-1 individuals compared with uninfected controls. This defective autophagic response was more pronounced in individuals with poor CD4 T-cell recovery, in which a positive correlation was observed between autophagy and CD4 T cell-count, establishing a link between autophagy and disease progression^15^.

In HIV-1 infected patients and animal models, autophagy dysregulation by HIV has been observed in cardiac tissue (decrease) and brain (increase), indicating an association between autophagy dysfunction and increased risk for HIV-1 related comorbidities^16,17^. Surprisingly, the effect of HIV on autophagy in lymphoid tissue is totally unknown, and in vivo, according to our knowledge, only one study examined autophagic levels in lymph nodes in HIV-1 infected individuals, showing higher autophagic levels in tissue of HIV-infected individuals compared with healthy donors^18^. As the evalution of the interplay between HIV and autophagy in vivo in lymphoid tissue is very difficult to determine, a more physiological model able to mimic human lymphoid organs in vitro is needed. Human lymphoid aggregated cultures (HLACs), prepared from tonsillar tissue maintains the cell populations and cytokine milieu found in vivo and thus, form an attractive primary cell model that displays robust HIV-1-mediated cytotoxicity and viral replication^19–21^. Given the interplay of HIV and autophagy and the central role that lymphoid tissue plays in HIV pathogenesis, this study was designed to investigate both the effect that the virus has on autophagy and the effect that the modulation of autophagy has on the virus in ex vivo lymphoid tissue cultures.

## Results

### Autophagy modulation on human lymphoid aggregate cultures (HLACs)

**Figure 1:**
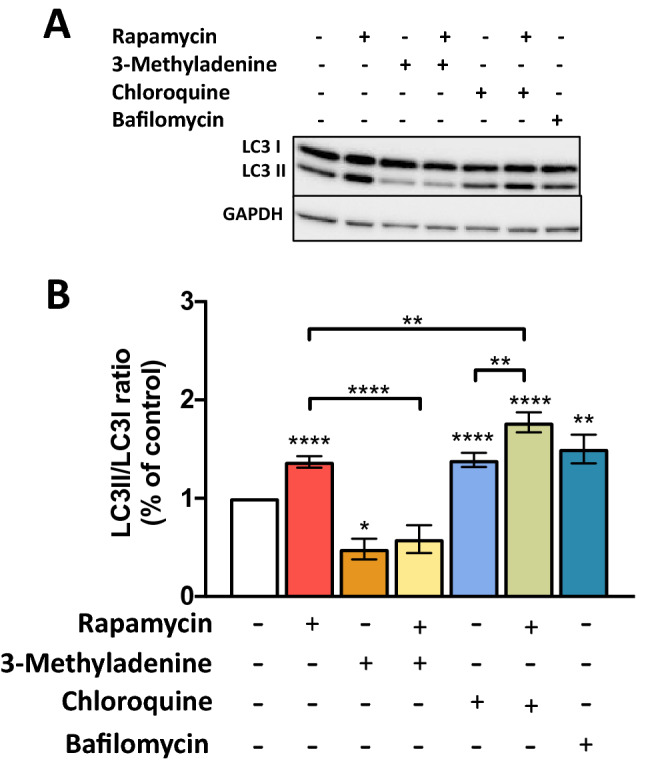
Basal autophagy and autophagy modulation and autophagic flux in HLACs. HLACs were treated for 24 h with medium or rapamycin (100 nM) and where indicated, 2 h pre-incubation with 3-methyladenine (10 mM) was done. Autophagic flux was analyzed by treating cells cultured with medium or rapamycin during 24 h and with chloroquine (30 µM) for the last 3 h. Cells cultured in medium were treated with bafilomycin (25 nM) for 3 h. The expression levels of LC3I, LC3II and GAPDH as loading control were assessed by western blotting. (**A**) Western blot image of a representative experiment. (**B**) LC3II/LC3I ratios are presented (mean ± SEM; n = 27 donors; for rapamycin + 3-methyladenine and 3-methyladenine alone n = 4 donors). Fold increase is relative to the untreated cells. Differences to the untreated control were tested by using one sample t-test. Differences between conditions were tested by t-test. *p < 0.05; **p < 0.01; ****p < 0.0001.

Basal autophagy, autophagy modulation and autophagic flux in HLACs was first analyzed using rapamycin, 3-methyladenine, chloroquine, and bafilomycin. In this model, a significant autophagy induction was observed after 24 h of rapamycin treatment, which resulted in an increase in LC3I and LC3II (Fig. 1A) and a higher LC3II/LC3I ratio compared to the untreated control (Fig. 1B and Suppl Fig. 1). A significant decrease in basal autophagy or in the rapamycin-induced autophagy was observed in the presence of 3-methyladenine. After chloroquine and bafilomycin treatment, an increase in the LC3II/LC3I ratio was observed after 3 h. Moreover, the LC3II/LC3I ratio was higher in cultures co-treated with rapamycin and chloroquine, indicative of the autophagic flux functionality in HLACs.

**Figure 2:**
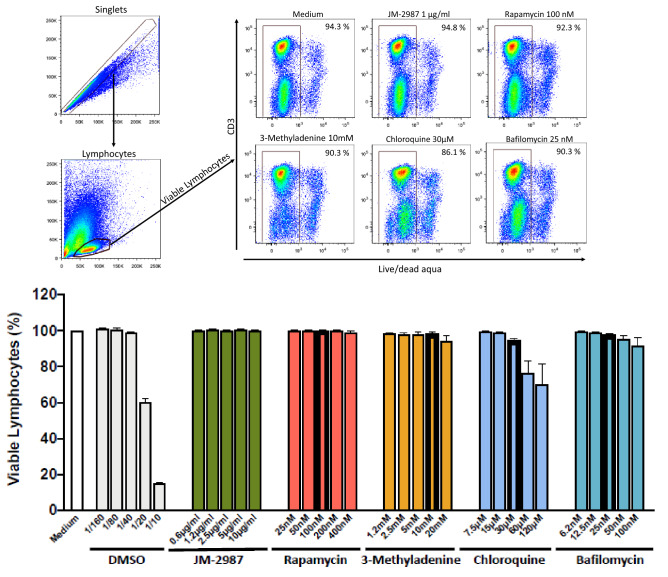
Autophagy modulators are not cytotoxic in HLACs. HLACs were treated with serial dilutions of rapamycin, 3-methyladenine, chloroquine, bafilomycin or medium for 3 days. The entry HIV inhibitor JM-2987 and DMSO were used as controls. Cell viability was measured by flow cytometry using a live/dead staining. (**A**) Gating strategy used. Doublets were excluded and lymphocytes were gated by forward and side scatter and live lymphocytes were selected. Representative flow cytometry plots are shown. (**B**) Proportion of viable lymphocytes following treatment with concentration ranges of different drugs (mean ± SEM; n = 4 donors, 2 of them measured in duplicate). Concentrations used throughout the study are highlighted in the graph in bold: 100 nM rapamycin, 10 mM 3-methyladenine, 30 µM chloroquine and 25 nM bafilomycin.

It has been previously described that autophagy modulators can be toxic to cells at relatively low concentrations^23^. Therefore, cytotoxicity was further analyzed in HLAC treated for 3 days with different concentrations of rapamycin, 3-methyladenine, chloroquine and bafilomycin. The percentage of viable cells was determined using a live/dead staining (Fig. 2). No significant decrease in cell viability was observed after 3 days of exposure with the concentrations of the drugs that we used to modulate autophagy (highlighted in bold in Fig. 2) or in any of the different concentrations tested of rapamycin, 3-methyladenine or bafilomycin. Chloroquine became cytotoxic at 60 µM and 120 µM but not at lower concentrations. The entry inhibitor JM-2987 and DMSO were used as a negative and positive controls, respectively.

### HIV infection induces autophagy in HLACs without an impairment in the response to autophagic modulators

**Figure 3:**
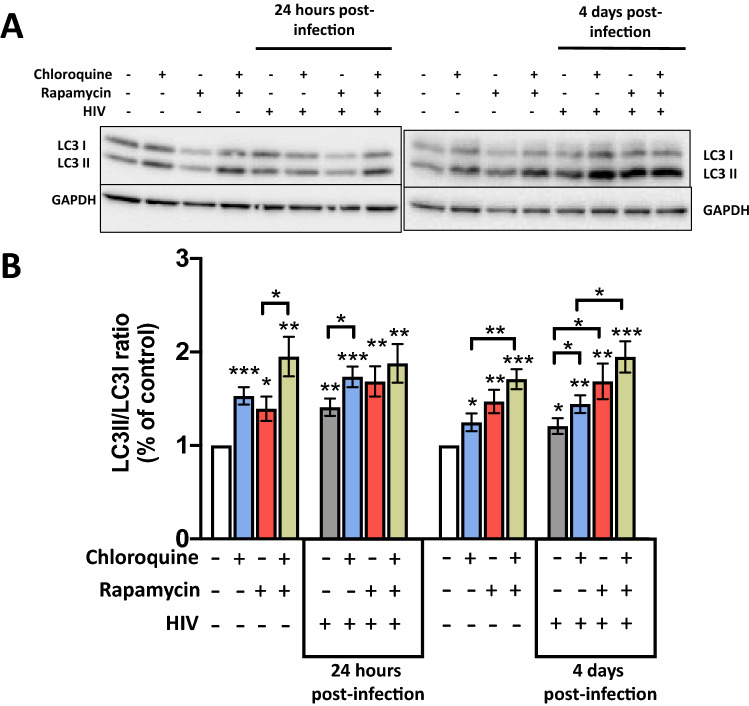
HIV-1 induces autophagy in HLACs without impairment in the response to autophagic modulators. HLACs were infected by spinoculation with a X4 strain of HIV-1. Three hours or 3 days after infection, and where indicated, cells were cultured for 24 h in the presence of rapamycin and/or chloroquine for the last 3 h. Cells were harvested and lysed and the expression level of LC3I, LC3II and GAPDH was revealed. (**A**) Western blot images of a representative experiment 24 h post-infection (3 h of infection + 24 h with rapamycin) and 4 days post-infection (3 days of infection + 24 h with rapamycin) are shown. Autophagic flux was analyzed by treating cells with chloroquine for the last 3 h of culture. (**B**) LC3I and LC3II levels were quantified and LC3II/LC3I ratios are presented (mean ± SEM; n = 9 donors). Fold increase relative to the untreated and uninfected controls. Differences to the control samples were tested by using one sample t-test. Differences between samples were tested by t-test. *p < 0.05; **p < 0.01; ***p < 0.001.

In order to investigate whether HIV-1 infection was able to modulate autophagy in lymphoid tissue, HLACs were infected and autophagy levels were analyzed 24 h and 4 days post-infection by western blot (Fig. 3A and Suppl Figs. 3 and 4). LC3II/LC3I ratio was significantly higher in HIV-infected HLACs (grey bars) than in uninfected cultures (white bars), both at 24 h and 4 days post-infection (Fig. 3B). Chloroquine (blue bars) and rapamycin (red bars) treatment led to an increase in the LC3II/LC3I ratio compared with infected HLACs without drugs (grey bars) (Fig. 3B), both 24 h and 4 days after infection. Rapamycin plus chloroquine (green bars) treatment resulted in a higher LC3II/LC3I ratio (Fig. 3), which indicates that infected cells have a functional autophagic flux. The autophagic modulation in uninfected HLACs cultured in parallel are shown for comparison with the infected ones.

### Autophagy modulation inhibits HIV-1 infection

**Figure 4:**
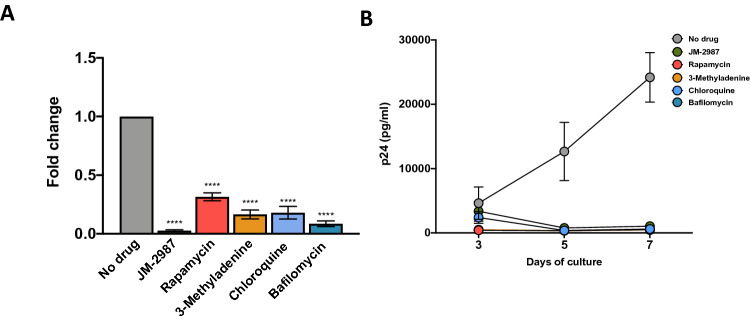
Autophagic modulation prevents HIV infection. (**A**) HLACs were pre-incubated for 2 h with rapamycin, 3-methyladenine, chloroquine and bafilomycin before infection by spinoculation with a X4 HIV-1 virus. The entry inhibitor JM-2987 was used as a control. Infections were maintained for 3 days and levels of total HIV DNA were measured by qPCR. Results are expressed as fold changes relative to the untreated infected control. Data represents mean ± SEM of 6–12 donors. Differences to the infected control were tested by using one sample t-test. ****p < 0.0001. (**B**) HLACs were pre-incubated for 2 h with rapamycin, 3-methyladenine, chloroquine and bafilomycin before infection with a X4 HIV-1 virus and kept in culture for 3 days. After 3 days, drugs were washed out, and medium was added to the culture for 4 more days. HIV p24 protein production was measured in the supernatant by ELISA at days 3, 5 and 7 post-infection. The entry inhibitor JM-2987 was used as a control. Shown are mean values ± SEM (n = 3).

To evaluate the effect of autophagy modulation on HIV-1 infection in HLACs, cultures were treated for 2 h with drugs, infected and then cultured in their presence for 3 days. Autophagy induction with rapamycin led to a significant decrease in total HIV DNA levels compared with the untreated infected control (Fig. 4A). Autophagy inhibition with 3-methyladenine, or blocking the autophagic flux, with chloroquine or bafilomycin, resulted also in a strong and significant decrease in the levels of total HIV DNA. JM-2987, added as a control, inhibited completely HIV DNA levels. In addition, after infection, a progressive increase over time in the p24 levels in the infected control without drug and an almost total inhibition with all tested drugs was observed (Fig. 4B).

### HIV-1 inhibition by autophagy modulation is envelope independent

**Figure 5:**
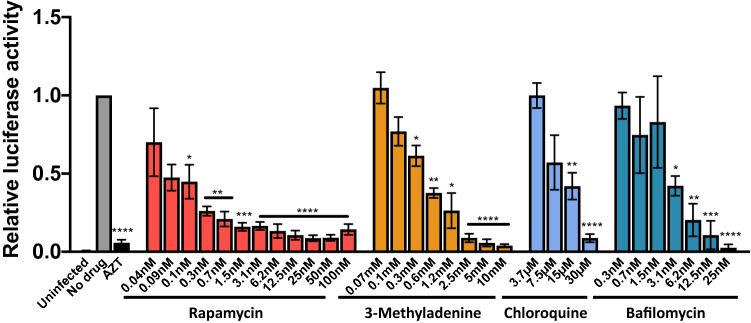
HIV envelope-independent inhibition after autophagy modulation. HLACs were pre-incubated for 2 h with serial dilutions (1:2) of rapamycin (100–0.049 nM), 3-methyladenine (10–0.078 mM), chloroquine (30–3.75 µM) and bafilomycin (25–0.195 nM) before infection. Cultures were infected by spinoculation with a (VSV-G)-NL4-3luc.R-E- HIV pseudovirus, and luciferase activity was measured 72 h after infection. The transcriptase inhibitor AZT (5 µg/ml) was used as a control. Results were expressed as luciferase activity relative to the infected control without drug. Data represents mean ± SEM of 4–6 donors each performed in duplicate. Differences to the control sample were tested by using one sample t-test. *p < 0.05; **p < 0.01; ***p < 0.001; ****p < 0.0001.

HLACs were pre-treated with serial dilutions of different autophagy modulators and infected with a single-cycle HIV-1 NL4-3 luciferase reporter pseudotyped virus with the heterologous Env vesicular stomatitis glycoprotein (VSV)-G. A significant decrease on luciferase activity was observed at a very wide range of rapamycin and 3-methyladenine concentrations compared to the untreated control (Fig. 5). With chloroquine and bafilomycin treatment, a significant decrease on luciferase activity was also detected, although the reduction was only significant at the highest concentrations (30 µM and 15 µM with chloroquine, and from 25 to 3.1 nM with bafilomycin) (Fig. 5). A total inhibition was detected in HLACs cultured with AZT. These results indicate that autophagy modulation is able to inhibit viral replication in an envelope independent manner.

### Autophagy modulation inhibit cell-to-cell HIV-1 transmission

**Figure 6:**
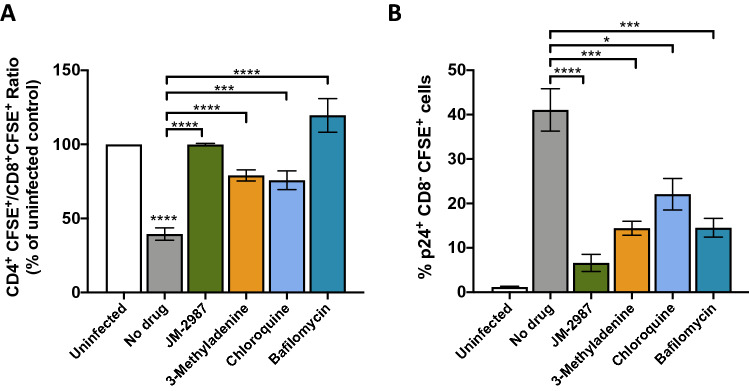
Inhibition of cell-to-cell HIV-1 transmission by autophagy inhibitors. HLACs were infected by spinoculation with a X4 HIV-1 virus for 3 days, and then cocultured in the presence of drugs with uninfected CFSE^+^-HLACs pre-treated for 2 h with JM-2987, 3-methyladenine, chloroquine and bafilomycin. (**A**) CD4^+^ T cell depletion results are represented as ratios of CD4^+^CFSE^+^/CD8^+^CFSE^+^ T-cells in untreated infected cultures or treated with different drugs compared to the uninfected control from the same donor (mean ± SEM; n = 6). (**B**) Frequency of intracellular p24 level in CD8^-^ CFSE^+^ cells are shown (mean ± SEM; n = 6).

To test the effect of autophagy inhibition in cell-to-cell transmission, we employed a co-culture approach, in which HLACs productively infected with HIV-1 were co-cultured with autologous carboxyfluorescein succinimidyl ester (CFSE)-labeled target tonsil cells pre-treated with autophagy inhibitors. Under these conditions, as expected, CFSE+-target tonsil CD4^+^ T cells were massively depleted when co-cultured with productively infected cells without drugs (Fig. 6A). However, cell depletion was significantly prevented after autophagy inhibition. The impact on HIV replication levels was evaluated measuring cell associated p24 antigen by flow cytometry (Fig. 6B). After infection, and in the absence of drugs, a significant increase in p24 levels was observed in CD8^−^ CFSE^+^ T cells compared to the uninfected control. CD8^-^ cells were analyzed to include CD4^+^ and CD4^−^ cells that are productively infected. In co-cultures, the presence of autophagic inhibitors resulted in a significant decrease in the intracellular p24 content in the CFSE^+^-target cells. A significant decrease in p24 levels was also observed with JM-2987.

### HIV-1 inhibition by mefloquine and quinacrine

**Figure 7:**
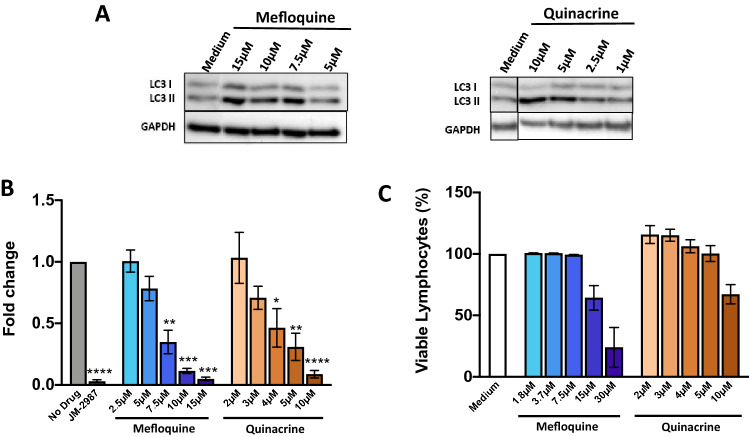
Autophagy modulation and HIV inhibition by mefloquine and quinacrine. HLACs were treated for 24 h with different concentrations of mefloquine, quinacrine or medium. Cells were harvested and lysates were immunoblotted for LC3I, LC3II and GAPDH. (**A**) Western blot image of one donor for each drug is presented. (**B**) HLACs were pre-incubated for 2 h with different concentrations of mefloquine, quinacrine or medium before infection. Cultures were infected by spinoculation with a X4 HIV-1 virus, and incubated for 3 days. Levels of total HIV DNA were measured by qPCR. The entry inhibitor JM-2987 was used as a control. Data represents mean ± SEM of 3 donors. Differences to the infected control without drug were tested by using one sample t-test. *p < 0.05; **p < 0.01; ***p < 0.001; ****p < 0.0001. (**C**) HLACs were treated with different concentrations of mefloquine, quinacrine or medium for 3 days. Cell viability was measured by flow cytometry using a live/dead staining. Doublets were excluded and lymphocytes were gated by forward and side scatter and live lymphocytes were selected. Proportion of viable lymphocytes following treatment (mean ± SEM; n = 2) are shown.

We next evaluated two other drugs currently used in the clinic: mefloquine and quinacrine. In agreement with previous publications^24,25^, both drugs were able to modulate autophagy in HLACs, and a dose dependent increase in LC3II expression was observed by western blot (Fig. 7A and Suppl Fig. 4). HLACs were exposed to different drug concentrations for 2 h, infected with a HIV-1 replicative virus and total HIV DNA levels were quantified after 3 days of infection. A significant HIV-1 inhibition was observed, measured as a dose-dependent decrease in HIV DNA levels with both tested drugs (Fig. 7B). No cytotoxicity was observed at concentrations with anti-HIV-1 activity (Fig. 7C).

## Discussion

Accumulating evidence suggests that autophagy plays a role in both HIV-1 replication and disease progression^26,27^. However, the cross talk between the virus and autophagy has been demonstrated using immortalized cell lines or exogenously activated primary cells. Nevertheless, none of these systems are representative of what is happening in vivo in an infected individual. Lymphoid organs are the primary sites of HIV replication and transmission, and is where the critical events of HIV disease progression occur^28^. HLACs recapitulate the biology of human lymphoid tissue more accurately than other in vitro models. In particular, cellular activation and cell diversity are preserved and even in the absence of exogenous activation the key in vivo pathogenic properties of HIV-1 infection, robust viral replication and CD4^+^ T cell depletion, are observed. In this study, HLACs displayed a fully functional autophagic activity; basal autophagy, induced response and autophagic flux were clearly observed. Thus, HLACs are a more physiological model to investigate HIV infection and its intricate relationship with autophagy.”.

In HLACs, a significant induction of autophagy was shown 24 h and 4 days after infection. HLACs are composed of a cellular mixture, so the observed induction is the global effect exerted by the virus in different cell types. In lymphoid tissue, a massive bystander cell depletion has been reported^19,29^. Therefore, it could be suggested that the observed autophagy could represent an Env-mediated autophagy induction in uninfected CD4^+^ T cells, as previously published^5,7^. However, we cannot rule out that there is induction in productively infected cells. In fact, the autophagic response could be different from the one observed in other in vitro infections, in which after PHA activation a much higher level of infection is reached. In addition, it is worthy to mention that the use of PHA already modulates autophagy^30^, which can complicate the interpretation of the effect of HIV in different in vitro systems. On the other hand, the effects that HIV infection exerts in vivo on the functionality of the autophagic pathway is barely known. In HLACs, we observed that both the autophagic response and the autophagic flux were preserved after infection. In HIV-1 infected individuals, we have previously reported a lower induced response and a lower autophagic flux than in uninfected controls when PBMCs were analyzed, with a high impairment in individuals who fail to recover their CD4^+^ T cells after ART treatment^15^. Furthermore, in PBMCs from HIV-1 infected LTNP, higher rapamycin-induced response and autophagic flux than individuals with normal progression has been shown^14^, which has led to establish a link between autophagy and disease progression. Importantly, our results are in agreement with in vivo data, where autophagy was up-regulated in axillary lymph nodes of HIV-1-infected individuals, compared to samples from uninfected donors^14^.

The therapeutic potential of autophagy inhibition, as a possible strategy to control HIV replication is supported by the observation that downregulation of autophagy-related genes and autophagy inhibition leads to in vitro HIV inhibition^7,8,11,31^. On the other hand, autophagy induction has been also associated with HIV-1 restriction in vitro in several experimental models^12,23^. However, reflecting the complex interaction between HIV and autophagy, autophagy induction and inhibition has been also associated with an increase in viral replication^7,11^. In ex vivo cultured lymphoid tissue, we have shown that autophagy modulation block cell-to-cell transmission and HIV infection, in an envelope-independent manner. The virus is not able to replicate whether the autophagic pathway is blocked or the pathway is hyperactivated.

Autophagy modulators are being clinically evaluated for treatment of several diseases, as everolimus to treat cancer, metformin in type 2 diabetes or rapamycin to prevent transplant rejection^32^. In HIV, the inhibition or hyperactivation of autophagy have been already used as an antiviral therapy. Rapamycin treatment at low concentrations was able to inhibit R5 HIV replication in a mouse model, and in HIV-infected kidney transplant recipients treatment was associated with a lower frequency of lymphocytes containing HIV DNA^33,34^. Similarly, chloroquine was used to treat HIV-infected individuals early in the HIV pandemic and a reduction in HIV-1 RNA load in plasma was reported, but the effect was not reproducible in other studies (Review in^35^). Very recently, the inhibition of autophagy during active HIV-1 infection is being considered as a potential supplementary treatment to effective ART for controlling HIV-1 infection^36^. Likewise, the induction of autophagy as a complement to a “block and lock” approach, designed to induce a state of deep viral latency, has been also lately proposed as a new strategy to achieve a functional HIV-1 cure (Review^37^).

We are aware of several study limitations. First, although we observed a significant increase of autophagy after HIV infection and inhibition after autophagy modulation, since autophagy has been measured by western blot, it has not been possible to establish whether the induction/inhibition is in productively infected or in uninfected bystander cells or which cell subtypes are affected. Autophagy can be detected by counting LC3 puncta using confocal microscopy, detecting double-membrane vesicles by transmission electron microscopy or by flow cytometry, however these assays have been used on fluorescent reporter constructs like GFP-LC3 in cell lines and are nor ideal to study mixed populations of primary cells like peripheral lymphocytes. In addition, we cannot rule out that the observed inhibition of HIV replication in HLACs with autophagy modulators is only due to their effect on autophagy since these drugs have an impact in multiple metabolic pathways^38,39^.

In conclusion, our results show that efficient HIV-1 replication requires a fine-tuned level of autophagy. Therefore, the autophagic pathway could be explored as a new target to treat HIV-1 infection. The modulation of cellular pathways instead of targeting the virus could be a potential approach to both, avoid resistance to antiretroviral treatment, and to treat individuals carrying resistant viruses.

## Material and methods

### Tonsils and generation of human lymphoid aggregate cultures (HLACs)

Tonsil tissue was removed from healthy donors. All individuals provided written informed consent, and the study was approved by the Ethics committee of the Hospital Germans Trias i Pujol (PI-15-125). Research was performed in accordance with the Declaration of Helsinki. Tissue was processed as previously described^19,20^. Briefly, tonsils were dissected and Human Lymphoid Aggregate Cultures (HLACs) were established in 96-well U-bottom plates (1 × 10^6^ cells/200 µL) and cultured at 37 °C in 5% CO_2_. Each figure legend provides details on how many individual donors were analyzed in the given experiment.

### Autophagy modulation in tonsil cells

HLACs were pre-treated with or without 3-methyladenine for 2 h and then incubated with rapamycin or medium, and cultured for 24 h at 37 °C. Bafilomycin and chloroquine were added to the culture for the last 3 h of incubation. For autophagy modulation with mefloquine and quinacrine, cells were treated with the drugs for 24 h at 37 °C. After incubation, cells were washed with PBS, and dry pellets were stored at − 80 °C. All modulators were purchased from Sigma-Aldrich (Spain).

### Western blot analysis

Dry pellets were lysed in 1X RIPA buffer (Werfen) supplemented with halt protease inhibitor cocktail (Invitrogen) and 1 mM phenylmethylsulfonyl fluoride (PMSF) (Werfen). Protein concentration was determined by Bradford assay (Thermo Fisher). Between 20 to 30 µg of protein were loaded in 12% SDS-PAGE gels (Thermo Fisher), transferred to 0.2 µm PVDF membranes (Bio-Rad Laboratories), blocked with 5% non-fat milk in PBS + 0.05% Tween-20 for 1 h at room temperature, and incubated overnight at 4 °C with primary antibodies: anti-LC3 (1:1000, NB100-2331, Novus Biologicals) and anti-GAPDH (1:5000, MA5-15738, Thermo fisher). After washing, membranes were incubated for 1 h at room temperature with secondary antibodies (1:10000 dilution, 115-036-071, Jackson ImunoResearch) and (1:5000, 711-036-152, Jackson ImunoResearch), revealed with SuperSignal West Pico Chemiluminescent Substrate (Thermo Fisher) and read through ChemiDoc Imaging systems (Bio-Rad). The same amount of protein was used among all different conditions of the same experiment. Bands density, corresponding to LC3I (16 kDa) and LC3II (14 kDa) forms of the LC3 protein, were quantified with Image Lab software.

### Cell viability assay

HLACs were treated with serial dilutions of drugs and incubated for 3 days at 37 °C. Viability was monitored by flow cytometry using a Live/Dead fixable Aqua cell staining kit (Invitrogen) and CD3 APC-Cy7 (Biolegend). Data was acquired in a LSRII cytometer and analyzed with FlowJo software.

### Autophagy analysis in HLACs after HIV infection

HLACs were infected by spinoculation (1200×*g* for 2 h at 4 °C) with 80 ng of p24 Gag of a HIV-1 X4-tropic strain and after spinoculation maintained 1–2 h at 37 °C without disturbing the pellet. Virus was removed after 3 h and cells were cultured for 24 h with rapamycin or 3 days with medium and then 24 h with rapamycin. Chloroquine was added to the cultures for the last 3 h of incubation. Uninfected HLACs were also treated in parallel with the infected ones and used as controls. Three replicates were done for each condition and, after incubation, replicates were pooled together. Cells were washed with PBS and dry pellets were stored at − 80 °C.

### Effect of autophagy modulation in HIV infection

HLACs were treated for 2 h with drugs and infected by spinoculation as explained before. The virus was washed out and fresh drugs were added. Cell cultures were maintained for 3 days at 37 °C and dry pellets were stored at − 80 °C until total HIV DNA analysis was performed.

In addition, HLACs were treated with drugs and infected for 2 h at 37 °C without spinoculation. The virus was washed out and fresh drugs were added. Cultures were maintained for 7 days at 37 °C. Supernatants were collected at days 3, 5 and 7 post-infection and the amount of p24 of HIV-1 capsid antigen was measured by AlphaLISA immunoassay (PerkinElmer).

### Total HIV DNA detection

DNA was isolated using Qiamp DNA Blood kit (Qiagen) according to the manufacturer’s instructions. Standard curves were generated by ten-fold serial dilutions (10^6^ to 10^1^) of recombinant plasmids expressing HIV-1 gag and the cellular gene CCR5. Quantitative real-time PCR was performed using a TaqMan Universal Master Mix (Applied Biosystems) and the primers and probes: 688F (5′-GACGCAGGACTCGGCTTG-3′); 809R (5′-ACTGACGCTCTCGCACCC-3′), F480PRO (5′-FAM-ACAGAGACACTTCCCGCCCCCG-TAMRA-3′) to detect HIV-1 gag and CCR5-576F (5′-TCATTACACCTGCAGCTCTCATTT-3′); CCR5-726R (5′-ACACCGAAGCAGAGTTTTTAGGAT-3′) and CCR5-661 T (5′-VIC-CTGGTCCTGCCGCTGCTTGTCA-TAMRA-3′) for CCR5 detection. Absolute HIV DNA copies per million cells were calculated for each sample and fold changes relative to the infected control without drug were determined. All real-time PCR reactions were performed on an ABI Prism 7000 (Applied Biosystems).

### HIV-1 pseudovirus production

Pseudotyped luciferase reporter viruses were prepared in HEK293T cells by co-transfection of pNL4-3luc.R-E- vector and the VSV-G envelope expression vector by using the X-tremGENE HP DNA Transfection Reagent (Sigma). Transfected cells were incubated for 48 h at 37 °C. Supernatant was harvested, treated with 100 U/mL of DNAse I (Invitrogen) for 1 h at 37 °C. Aliquots were stored at − 80 °C until use.

### Single-cycle infectivity assay

HLACs were incubated for 2 h at 37 °C with serial dilutions of drugs and then infected with the (VSV-G)-NL43 luciferase reporter pseudovirus by spinoculation as described before. After washing out the virus, cells were cultured for 3 days in the presence of fresh drugs. Cells were lysed with Britelite plus Reporter Gene assay (PerkinElmer) and luciferase was measured using an EnSight multimode plate reader with the Kaleido software.

### Analysis of cell-to-cell HIV transmission

HLACs were infected with a X4 virus by spinoculation as explained before. After 3 days, uninfected cells were labeled with CellTrace CFSE (2 µM, Invitrogen) for 5 min at room temperature. Uninfected-CFSE^+^ (0.6 × 10^6^ cells) were pre-treated for 2 h with or without autophagy inhibitors at 37 °C. Then, infected or uninfected CFSE^-^ cells were added to the treated CFSE^+^ target cells in a ratio 1:1, incubated in the presence or absence of drugs for 2 days at 37 °C, collected and analyzed by flow cytometry. Cells were stained with the antibodies CD3-APC-Cy7, CD4-APC and CD8-PerCP (BioLegend), fixed and permeabilized (FIX & PERM kit, Thermo Fisher) and stained with anti-p24 antibody (KC57-PE, Coulter). To calculate CD4^+^ T cell depletion, total live lymphocytes were first gated according to forward and side scatter and then gated on the CD3^+^ population. Next, CFSE^+^ cells were selected, CD4^+^ and CD8^+^ T cells were gated and CD4^+^/CD8^+^ ratio was obtained. Cell depletion was calculated as a percentage compared to the uninfected control as previously described^22^. To analyze intracellular p24 level, CD3^+^ CFSE^+^ cells were gated and CD8-negative cells were selected to include both CD4-positive and CD4-negative cells. Data was acquired in a LSRII cytometer and analyzed with FlowJo software.

### Statistical analysis

Statistical analysis and data visualization were performed with GraphPad Prism (version 9). One sample t-test and two-tailed unpaired Student’s t test were used. Differences were considered significant at *p < 0.05, **p < 0.01, ***p < 0.001 and ****p < 0.0001.

## Supplementary Information

Supplementary Legends.

Supplementary Figures.
