# Author Correction: Modulation of the autophagic pathway inhibits HIV-1 infection in human lymphoid tissue cultured ex vivo

**DOI:** 10.1038/s41598-023-30114-z

**Published:** 2023-02-20

**Authors:** Sònia Pedreño‑López, Elisabet García, Dolores Guerrero, Elisabet Gómez‑Mora, Laura Molina Mateu, Fernando Orera Pérez, Jordi Senserrich, Bonaventura Clotet, Cecilia Cabrera

**Affiliations:** 1 AIDS Research Institute‑IrsiCaixa and Health Research Institute Germans Trias i Pujol (IGTP), Hospital Germans Trias i Pujol, Universitat Autònoma de Barcelona, Carretera del Canyet S/N, 08916 Badalona, Barcelona Spain; 2 Otorhinolaryngology Department, Hospital Germans Trias i Pujol, Universitat Autònoma de Barcelona, 08916 Badalona, Spain; 3 Infectious Diseases Department, Hospital Germans Trias i Pujol, Badalona, Catalonia Spain; 4 Universitat de Vic Central de Catalunya, Vic, Catalonia Spain

Correction to: *Scientific Reports* https://doi.org/10.1038/s41598-022-11181-0, published online 06 May 2022

The original version of this Article contained an error in the Funding section.

“Cecilia Cabrera is a researcher at the Fundació Institut de Recerca en Ciències de la Salut Germans Trias i Pujol supported by the Health Department of the Catalan Government/Generalitat de Catalunya and ISCIII. Work in our laboratory has been funded in part by grants from Instituto de Salud Carlos III, Fondo de Investigación Sanitaria (FIS) PI12/02408, PI15/01053 cofinanced by FEDER and by Grifols.”

now reads:

“Cecilia Cabrera is a researcher at the Fundació Institut de Recerca en Ciències de la Salut Germans Trias i Pujol supported by the Health Department of the Catalan Government/Generalitat de Catalunya and ISCIII. Work in our laboratory has been funded in part by grants from Instituto de Salud Carlos III, (PI12/02408, PI15/01053, PI18/01226), co-funded by ERDF, "A way to make Europe" and by Grifols.”

The original Article has been corrected.

