# Supplementary material for: Modulation of the autophagic pathway inhibits HIV-1 infection in human lymphoid tissue cultured ex vivo
Source: Sci Rep. 2022 May 6;12:7439. doi: 10.1038/s41598-022-11181-0 (PMC9076641; doi:10.1038/s41598-022-11181-0)
Supplement: Supplementary file 1 — Supplementary Legends. [file 41598_2022_11181_MOESM1_ESM.docx]

**Supplementary Figure 1.** Contains full scan of uncropped western blot corresponding to Figure 1A.

**Supplementary Figure 2 and Supplementary Figure 3.** Full scans of uncropped blots with different exposures corresponding to Figure 3A.

**Supplementary Figure 4.** Full scans of uncropped blots with different exposures corresponding to Figure 7A.
