# Supplementary material for: Modulation of the autophagic pathway inhibits HIV-1 infection in human lymphoid tissue cultured ex vivo
Source: Sci Rep. 2022 May 6;12:7439. doi: 10.1038/s41598-022-11181-0 (PMC9076641; doi:10.1038/s41598-022-11181-0)

Suppl Figure 1.

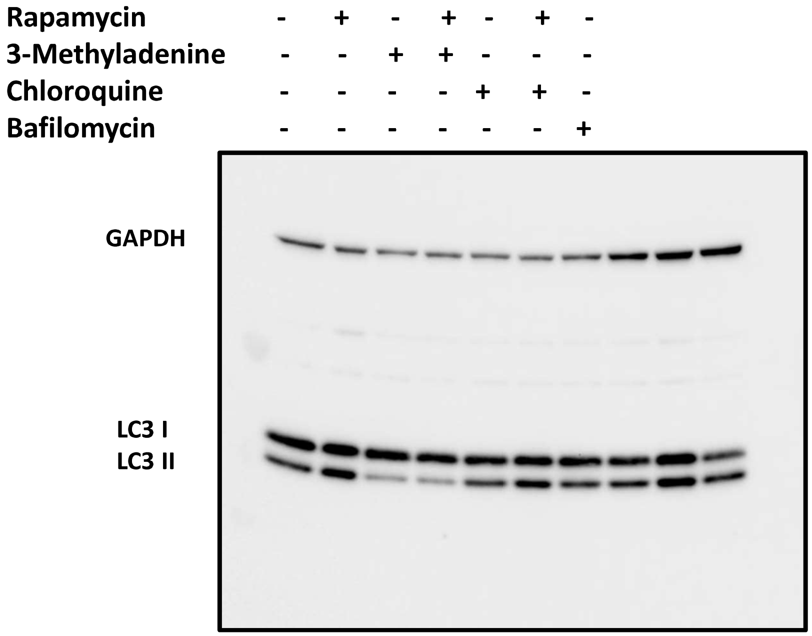

Full scan Figure 1.

Suppl Figure 2. **Full scan Figure 3.**

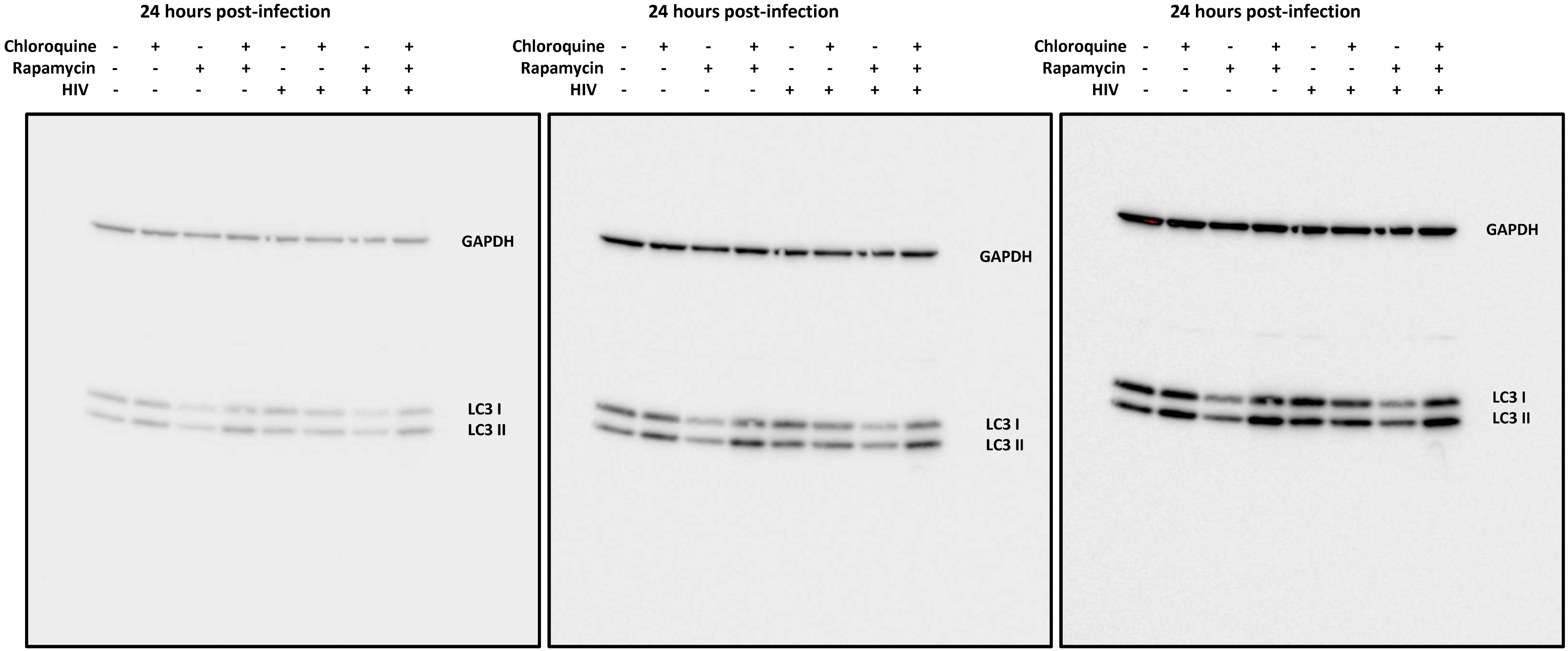

Low exposure Medium exposure High exposure

Suppl Figure 3.

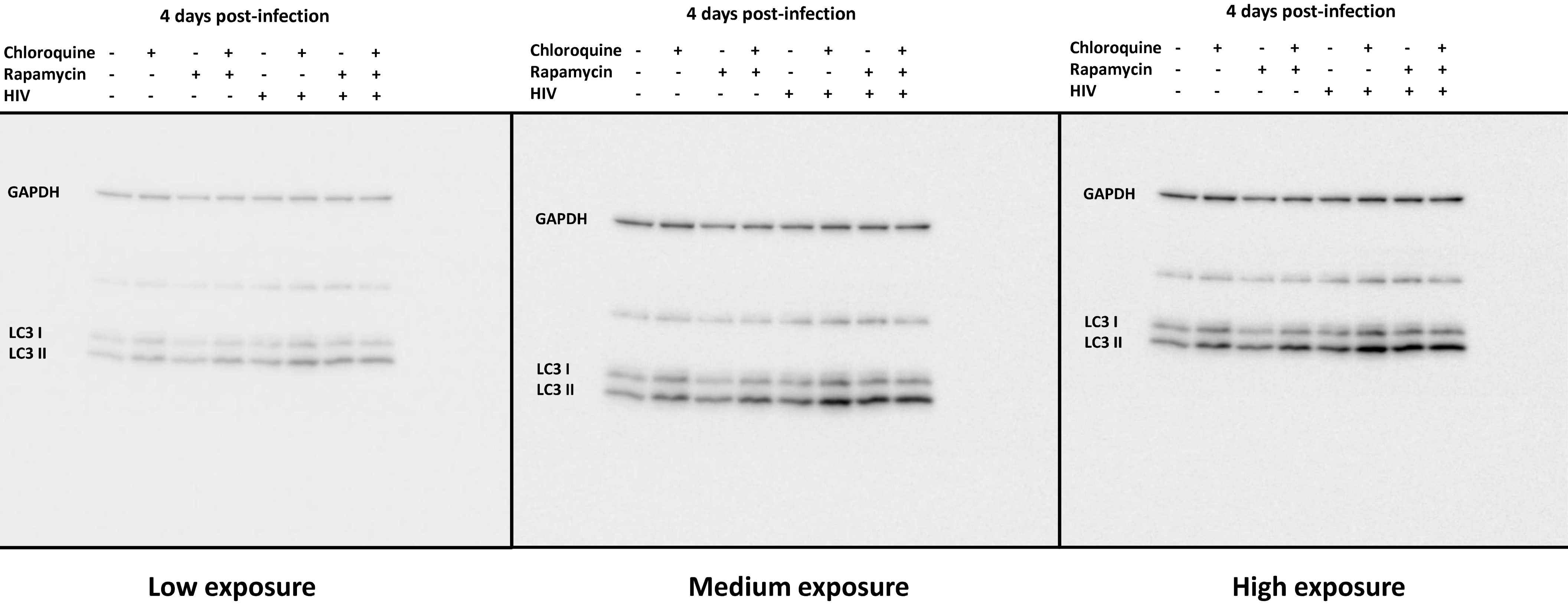

Full scan Figure 3.

Suppl Figure 4.

Full scan Figure 7.

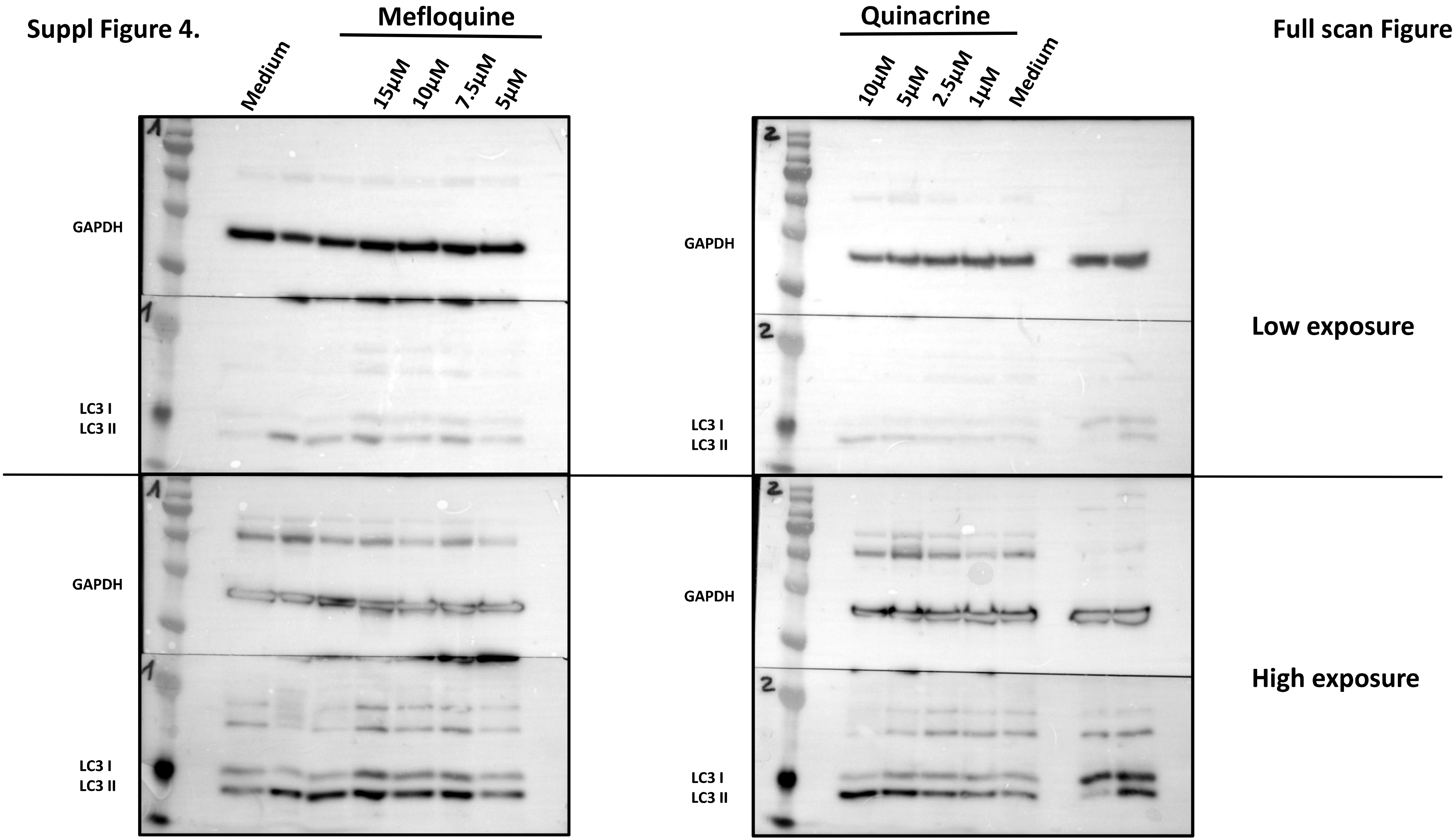

Supplement: Supplementary file 2 — Supplementary Figures. [file 41598_2022_11181_MOESM2_ESM.pdf]
